# Data leakage in deep learning studies of translational EEG

**DOI:** 10.3389/fnins.2024.1373515

**Published:** 2024-05-03

**Authors:** Geoffrey Brookshire, Jake Kasper, Nicholas M. Blauch, Yunan Charles Wu, Ryan Glatt, David A. Merrill, Spencer Gerrol, Keith J. Yoder, Colin Quirk, Ché Lucero

**Affiliations:** ^1^SPARK Neuro Inc., New York, NY, United States; ^2^Neuroscience Institute, Carnegie Mellon University, Pittsburgh, PA, United States; ^3^Pacific Brain Health Center, Pacific Neuroscience Institute and Foundation, Santa Monica, CA, United States; ^4^Saint John's Cancer Institute at Providence Saint John's Health Center, Santa Monica, CA, United States; ^5^Psychiatry and Biobehavioral Sciences, Semel Institute for Neuroscience and Human Behavior, David Geffen School of Medicine at University of California, Los Angeles, Los Angeles, CA, United States

**Keywords:** electroencephalography, deep neural networks, data leakage, cross-validation, Alzheimer's disease, epilepsy

## Abstract

A growing number of studies apply deep neural networks (DNNs) to recordings of human electroencephalography (EEG) to identify a range of disorders. In many studies, EEG recordings are split into segments, and each segment is randomly assigned to the training or test set. As a consequence, data from individual subjects appears in both the training and the test set. Could high test-set accuracy reflect data leakage from subject-specific patterns in the data, rather than patterns that identify a disease? We address this question by testing the performance of DNN classifiers using segment-based holdout (in which segments from one subject can appear in both the training and test set), and comparing this to their performance using subject-based holdout (where all segments from one subject appear exclusively in either the training set or the test set). In two datasets (one classifying Alzheimer's disease, and the other classifying epileptic seizures), we find that performance on previously-unseen subjects is strongly overestimated when models are trained using segment-based holdout. Finally, we survey the literature and find that the majority of translational DNN-EEG studies use segment-based holdout. Most published DNN-EEG studies may dramatically overestimate their classification performance on new subjects.

## 1 Introduction

Translational neuroscience studies increasingly turn to deep neural network (DNN) models to find structure in neural data. The power of DNN models comes from their ability to discover patterns in the data that researchers would not have been able to specify. DNN classifiers have the potential to revolutionize medical care by increasing the speed, accuracy, and availability of diagnosis (Mall et al., 2023). DNNs have been trained on a variety of imaging techniques to identify a wide range of clinical conditions. Many of these studies use DNNs to diagnose diseases based on anatomical neuroimaging. For example, DNN models can identify Alzheimer's disease (AD) using structural magnetic resonance imaging (MRI) (Wen et al., 2020), and a variety of cancers and brain injuries using CT scans (Hosny et al., 2018; Kaka et al., 2021). In addition to anatomical data, a large number of studies have used DNNs to identify diseases from functional neuroimaging data. For example, DNNs with functional MRI show promise for identifying AD, Autism spectrum disorders, attention-deficit/hyperactivity disorder (ADHD), and schizophrenia (Wen et al., 2018). Furthermore, DNNs have been used with electroencephalography (EEG) to study a variety of different neural and cognitive disorders (de Bardeci et al., 2021).

Deep learning helps to reveal previously-unknown patterns in neuroimaging data, but it also presents researchers with subtle pitfalls. One set of challenges concerns how the data are split into separate training and test sets. The training set is used to fit the model's parameters, and the test set is used to estimate the model's performance on new data (a third subset of the data is often held aside as a validation set, used to tune the model's hyperparameters and to determine when to stop training the model). In some cases, researchers train their model on one subset of the available data, and then evaluate the model's performance on a separate test set. In other cases, researchers use cross-validation (CV) to train and test models on multiple subsets of the data. Under both of these approaches, researchers must be careful to avoid “data leakage” when splitting the data into training and test sets. Data leakage, which arises when information about the test set is present in the training set, results in a positively-biased estimate of the model's performance (Kaufman et al., 2012). For example, in a data-mining competition focused on identifying patients with breast cancer, one team of researchers found that the patient ID number carried predictive information about cancer risk (Rosset et al., 2010). These ID numbers may have appeared after compiling data from different medical institutions. Because the ID number was assigned based on patients' diagnosis, it constitutes a source of data leakage (Rosset et al., 2010). In general, data leakage occurs when an experimenter handles the data in a way that artificially introduces correlations between the training and test sets.

DNN models typically require a large amount of training data to perform well, but neural datasets are usually expensive and difficult to obtain. To increase the number of observations available to train the model, these studies often split a single neural recording into multiple samples, and use each sample as a separate observation during training or testing. For example, a 3D structural MR volume could be split into multiple 2D slices, and an fMRI time-series could be split into multiple segments of time (Wen et al., 2020). When multiple observations from a single subject are included in both the training and test sets, it constitutes data leakage: Instead of learning a generalizable pattern, these models could learn characteristics of the individual subjects in the training set, and then simply recognize those familiar subjects in the test set. As a result, these models perform well in the study's test set, leading the researchers to believe they have a robust classifier. In new subjects, however, the model may fail to generalize.

Prior research has shown that leakage of subject-specific information—sometimes referred to as “identity confounding” (Chaibub Neto et al., 2019)—occurs in a number of different research areas. For example, this type of data-leakage occurs in published MRI studies (Wen et al., 2020). Furthermore, leakage of subject-specific information is widespread in translational studies using optical coherence tomography (OCT), and leads to strongly inflated estimates of test accuracy (Tampu et al., 2022). Identity confounding has also been demonstrated in studies that make clinical predictions on the basis of smartphone data, wearable sensor data, and audio voice recordings (Saeb et al., 2017; Tougui et al., 2021).

Studies using DNNs with EEG are particularly susceptible to data leakage. In these studies, each subject's full EEG time-series (lasting several minutes) is commonly divided up into brief segments (lasting several seconds) (de Bardeci et al., 2021). Each segment is then used as a separate observation during training or testing. This segmentation procedure is meant to ensure that DNN models have enough training data to learn robust representations of the patterns that characterize a disease, and to prepare the data for commonly-used model architectures. However, EEG segmentation leads to data leakage if the same subjects appear in both the training and test sets. Segments of EEG from one subject are more similar to each other than to segments from different subjects (Demuru and Fraschini, 2020). Instead of learning an abstract representation that would generalize to new subjects, a DNN model could therefore achieve high classification accuracy by associating a label with each subject's idiosyncratic pattern of brain activity. As a consequence, randomly splitting EEG segments into training and test sets results in data leakage, and a biased estimate of test performance: accuracy is high on the researchers' test set, but the classifier will generalize poorly to new subjects. In a clinical setting, this leads to an apparently-promising diagnostic tool that fails when applied to new patients. To avoid this kind of data leakage, all segments from a given subject must be assigned to only a single partition of the data (i.e., train *or* validation *or* test).

How does leakage of subject-specific information bias the results of translational DNN-EEG studies? Here we address this question by examining the effects of data leakage in two case studies, and then reviewing the published literature to gauge the prevalence of this leakage. In the case studies, we reproduce two convolutional neural network (CNN) architectures used by published studies—both of which used a train-test split that introduced data leakage. In order to focus on the ways in which leakage results from the train-test split, and to facilitate comparison with prior literature, we reuse these published model architectures without any modification. First, we use a CNN to classify subjects as either healthy or as having dementia due to Alzheimer's disease. Second, we use a CNN to classify whether segments of time contain an epileptic seizure. In both datasets, we find that real-world performance is dramatically overestimated when data from individual subjects is included in both the training and test sets. In the literature review, we find that the majority of translational DNN-EEG studies suffer from data leakage due to data from individual subjects appearing in both the training and test sets.

## 2 Method

### 2.1 Deep neural network analysis overview

To investigate how segment-based holdout leads to data leakage, we reproduced the model architectures from two published studies (Oh et al., 2020; Rashed-Al-Mahfuz et al., 2021). The goal of these analyses was not to develop an optimal architecture, but rather to evaluate the impact of different cross-validation choices on the estimated model performance. We therefore re-used the published architectures and data processing pipelines without modification, and without any model selection or hyperparameter tuning. The code necessary to reproduce both of these DNN models is provided in the Supplementary material.

### 2.2 Experiment 1: Alzheimer's disease diagnosis

#### 2.2.1 EEG data

We analyzed EEG data that was collected for a previously published study (Ganapathi et al., 2022). These EEG recordings were provided to us by the Pacific Neuroscience Institute. All procedures were approved by the St. John's Cancer Institute Institutional Review Board (Protocol JWCI-19-1101) in accordance with the Helsinki Declaration of 1975. Patients were evaluated by a dementia specialist as part of their visit to a specialty memory clinic (Pacific Brain Health Center in Santa Monica, CA) for memory complaints. This evaluations included behavioral testing as well as EEG recordings. After these evaluations, subjects were selected by retrospectively reviewing charts for patients aged 55 and older seen between July 2018 and February 2021.

Patients received a consensus diagnosis from a panel of board-certified dementia specialists. Diagnoses were performed using standard clinical methods on the basis of neurological examinations, cognitive testing (MMSE Folstein et al., 1975 or MoCA Nasreddine et al., 2005), clinical history (e.g., hypertension, diabetes, head injury, depression), and laboratory results (e.g., vitamin B-12 levels, thyroid stimulating hormone levels, and rapid plasma regain testing). These tests were used to rule out reversible causes of memory loss and to diagnose subjective cognitive impairment (SCI), mild cognitive impairment (MCI), and dementia. EEG data was not included in the diagnostic process. Cognitive impairment was diagnosed on the basis of MMSE [or MoCA scores converted to MMSE (Bergeron et al., 2017)], with MCI diagnosed according to established criteria (Langa and Levine, 2014). MCI was distinguished from dementia on the basis of preserved independence in functional abilities, and a lack of significant impairment in social or occupational functioning. SCI was diagnosed in patients with subjective complaints but without evidence of MCI. Diagnostic categorization was based on the clinical syndromes (Langa and Levine, 2014), and did not consider disease etiology or subtypes within each stage.

EEG data were recorded at 250 Hz using the eVox System (Evoke Neuroscience), with a cap that included 19 electrodes following the International 10-20 system (FP1, FP2, F7, F3, Fz, F4, F8, T7, C3, Cz, C4, T8, P7, P3, Pz, P4, P8, O1, and O2). The full EEG session included a 5-min block of eyes-open rest, a 5-minute block of eyes-closed rest, and a 15-min go/no-go task. In this study, we analyzed only the eyes-open resting-state data. Recordings were low-pass filtered below 125 Hz, and split into non-overlapping segments of 2 s (500 samples) for model training. Channels were stacked to produce matrices of shape (500, 19) as model inputs.

We selected all 49 subjects in the dataset who were diagnosed with dementia due to Alzheimer's disease (18 male, 31 female; age 73.9 ± 6.8 years). As a comparison, we selected an equal number of subjects with subjective cognitive impairment (SCI; *n* = 49, 18 male, 31 female; age 63.9 ± 11.4 years).

#### 2.2.2 Architecture

Because our goal was to evaluate the effects of different cross-validation strategies on generalizability, we re-used a previously-published model architecture without modification. We reproduced the model architecture from Oh et al. (2020); this model is a 1D convolutional neural network trained to classify segments of time-series EEG data as SCI or AD.

This model learns temporal filters that are applied equivalently across each EEG channel. Progressing through the network, subsequent layers build more complex features that take into account a larger temporal receptive field, and some invariance is achieved through pooling over time. The model consisted of four convolutional layers, each followed by rectification, max pooling, and batch normalization; convolutional layers were followed by two dense fully-connected layers of 20 and 10 hidden units, respectively, each rectified, and finally a dense connectivity to the output layer with 2 units representing AD yes/no probability logits. All deep learning models were trained with Keras and Tensorflow. The exact Keras code used to specify the architecture can be found in the Supplementary material.

#### 2.2.3 Training

Models were trained for 70 epochs without any early stopping or hyperparameter tuning. A batch size of 32, initial learning rate of 0.0001, and the Adam optimizer were used to optimize models. Training accuracy was computed and stored online during each epoch, and averaged across batches to report the training accuracy for each epoch. To visualize how quickly the models reached their final performance, test set accuracy was also computed after each epoch, averaged across batches. Since we reused the model architecture from prior published work, no model selection was performed; performing ongoing validation on the test is therefore not a source of data leakage. For segment-based holdout, data were split using 10-fold cross-validation (see “Cross-validation” for details).

### 2.3 Experiment 2: seizure detection

#### 2.3.1 EEG data

We analyzed data from the Siena Scalp EEG Database (Detti, 2020; Detti et al., 2020) hosted on PhysioNet (Goldberger et al., 2000). These recordings were collected in accordance with the Declaration of Helsinki, and approved by the Ethical Committee of the University of Siena. Participants provided written informed consent before beginning data collection. This dataset includes recordings from 14 epilepsy patients (age 20–71 years, nine male) digitized at 512 Hz with electrodes arranged following the International 10-20 system. Seizures in the data were labeled by an expert clinician. This dataset contains 47 seizures in ~128 h of recorded EEG. To ensure that the data were balanced between seizure and non-seizure epochs, we selected non-seizure data from the beginning of each subject's recordings to match the duration of their seizure-labeled data. This led to 47 min 21 s of data in each condition (1 h 34 min 42 s in total).

In contrast to the previous section where raw time series were used, EEG data were prepared for the classifier analysis in the frequency domain, following the approach used by Rashed-Al-Mahfouz and colleagues (Rashed-Al-Mahfuz et al., 2021). Spectrograms were computed with a window length of 256 samples (0.5 s) overlapping by 128 samples (0.25 s), using a Hann taper. Spectrograms were then divided into segments of 1.5 s. As in the original study, we used the RGB representation of the spectrogram (viridis color-map), and exported as 224 × 224 × 3 images for training and testing with the CNN models.

#### 2.3.2 Architecture

The aim of this study was to evaluate the impact of different cross-validation choices, not to identify a highly-performing model architecture. We therefore reused the model architecture presented by Rashed-Al-Mahfuz et al. (2021) without modification. No model selection or hyperparameter tuning was performed. To handle 3D spectrogram data (vs. 2D time-series used in the previous section), a 2D convolutional neural network was used. This model learns 2D spectrotemporal features that are applied equivalently across the spectrogram. The model contains four convolutional layers, each followed by rectification, pooling, and batch normalization, followed by two hidden fully-connected layers of 256 and 512 units each, dropout, and a final classification layer of 2 units corresponding to seizure yes/no. The exact Keras code used to specify the architecture can be found in the Supplementary material.

#### 2.3.3 Training

Models were trained for 70 epochs with no early stopping. We used the RMSProp optimimzer with a batch size of 32 and a learning rate of 0.00001. Training accuracy was computed and stored online during each epoch, and averaged across batches to report the training accuracy for each epoch. To visualize how quickly the models reached their final performance, test set accuracy was also computed after each epoch, averaged across batches. Since we reused the model architecture from prior published work, no model selection was performed; performing ongoing validation on the test is therefore not a source of data leakage.

### 2.4 Cross-validation

**Figure 1:**
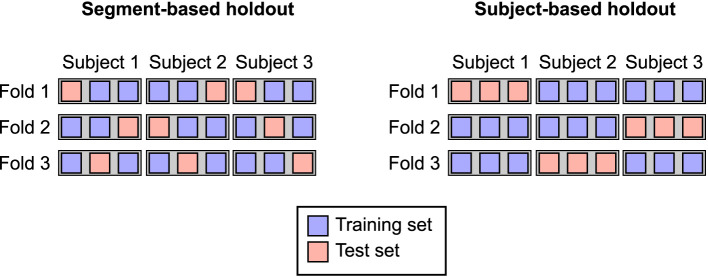
Illustration of segment-based and subject-based holdout. This example shows cross-validation with three participants, each of whom have three segments of data, and 3-fold cross-validation (CV). Each row shows a separate CV fold. Each square illustrates a single EEG segment, with blue squares indicating observations in the training set and red squares indicating observations in the test set. Gray rectangles are drawn around observations from the same subject.

This study is primarily concerned with the consequences of different approaches to splitting the data between training and test sets. We assess two types of train-test split: (1) holding out individual segments of EEG data without regard for subject ID (“segment-based holdout”), and (2) holding out entire subjects, ensuring that all segments for a given subject appear in only the training or the test set (“subject-based holdout”; Figure 1).

#### 2.4.1 Segment-based holdout

Segment-based cross-validation considers all EEG segments to be equivalent, and divides them into training and validation partitions without considering subject ID. This segment-holdout approach will lead to data leakage if there is statistical non-independence due to multiple EEG segments coming from each subject. Given *n* segments and *m* time-points per segments, we construct a matrix *X* of EEG segments of size (*n, m*), and a vector ***y*** of diagnostic label of length *n*. The cross-validation is a simple partition of the index vector ***α*** = {1, 2, …, *n*} into disjoint subsets ***α***_train_ and ***α***_test_. Where *X*_*i*_ gives the *i*th segments of *X*, we then have *X*_train_ = {*X*_*i*_}∀*i* ∈ ***α***_train_, *X*_*test*_ = {*X*_*i*_}∀*i* ∈ ***α***_test_, and ***y***_train_ = {***y***_*i*_}∀*i* ∈ ***α***_train_, ***y***_test_ = {***y***_*i*_}∀*i* ∈ ***α***_test_.

#### 2.4.2 Subject-based holdout

Subject-based cross-validation takes into account which subject each EEG segment comes from. This approach enforces that each subject appears in only one partition of the cross-validation, ensuring there is no leakage of subject-level information across training and test sets. To create this split, we consider an additional subject vector ***s***, which is used to constrain the partition of *X* and ***y***. Concretely, rather than partitioning the index vector ***α***, we partition the unique subject vector ***s***_*u*_, which gives the unique entries of ***s***, and collect all corresponding segments from each subject contained in train and validation partitions into ***α***_train_ and ***α***_test_. This enforces the constraint that ***s***_*i*_≠***s***_*j*_∀*i* ∈ ***α***_train_, *j* ∈ ***α***_test_. To perform k-fold cross-validation, we first divide ***s***_*u*_ into *k* non-overlapping chunks, and each chunk to serve as the validation data in each fold of cross-validation, where the remaining *k*−1 chunks are reserved for training.

### 2.5 Literature review

We searched the literature for studies that used deep learning with segments of EEG to classify a variety of diseases. We searched Google Scholar for papers investigating Alzheimer's disease, Parkinson's disease, attention-deficit/hyperactivity disorder (ADHD), depression, schizophrenia, and seizures. We then searched the references of these papers to identify additional publications for inclusion. Following this search, we included every study that used a DNN to identify psychiatric or neurological conditions using EEG. This non-exhaustive search included 63 papers, all of which were published since 2018 and used deep learning to study one of the conditions named above.

Next, we examined how the training and test sets were determined in these studies. If a paper specified that the EEG recordings were split into segments, but did not specify that they used subjects as an organizing factor of the train-test split, we labeled that study as using “segment-based” holdout. Some papers specifically stated that segments from individual subjects were included in both the training and test sets (for example, studies that trained separate models for each subject); these studies were also labeled as segment-based holdout. If a paper specified that all the segments from a single subject were assigned to only the training or the test set, we labeled that study as using “subject-based” holdout. If a study used both segment-based and subject-based holdout in different analyses, we labeled the study as “both”. We labeled studies as “unclear” if we could not determine whether the models were trained on segments of EEG recordings, and it was not explicitly stated that subjects were used as a factor in the holdout procedure.

## 3 Results

### 3.1 Data leakage leads to biased test-set accuracy

We analyze two datasets to test how the estimated accuracy of a DNN classifier depends on the train-test split. First, we examine the effects of data leakage in a patient-level classifier by training a model to diagnose Alzheimer's disease. Second, we examine the effects of data leakage in a segment-level classifier by training a model to identify periods of time that include an epileptic seizure. In each of these analyses, we reuse a published DNN architecture to analyze an existing dataset.

#### 3.1.1 Identifying patients with Alzheimer's disease

**Figure 2:**
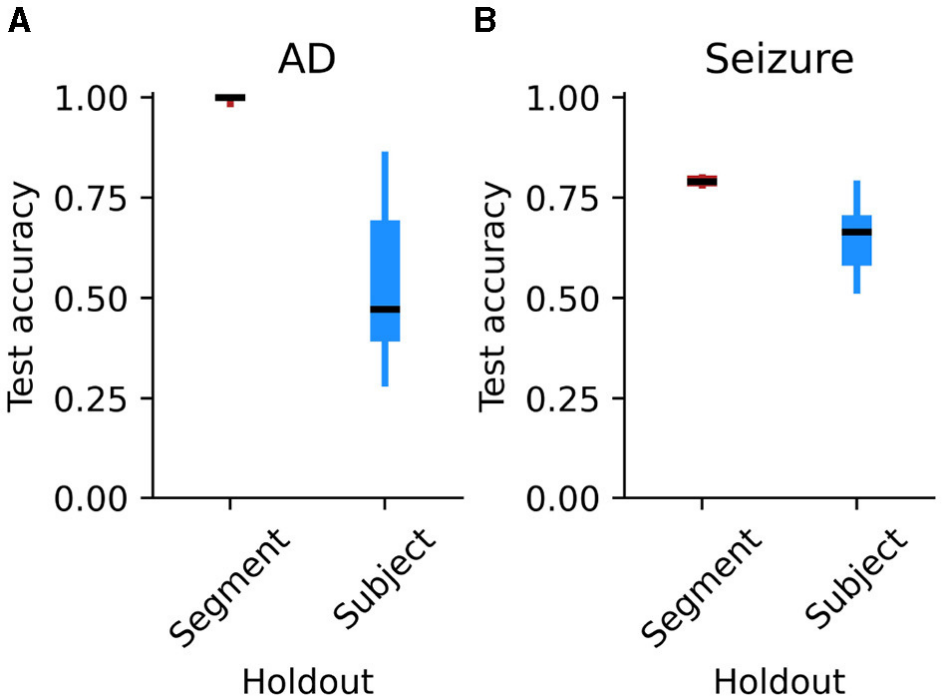
Test-set accuracy of CNN models predicting held-out data, plotted separately for segment-based holdout and subject-based holdout. **(A)** Accuracy for models trained to classify Alzheimer's disease in individual subjects. Boxes show the inter-quartile range, dark lines show the median, and whiskers extend to the minimum and maximum points. **(B)** Accuracy for models trained to identify seizures in segments of EEG data. Details as in **(A)**.

**Figure 3:**
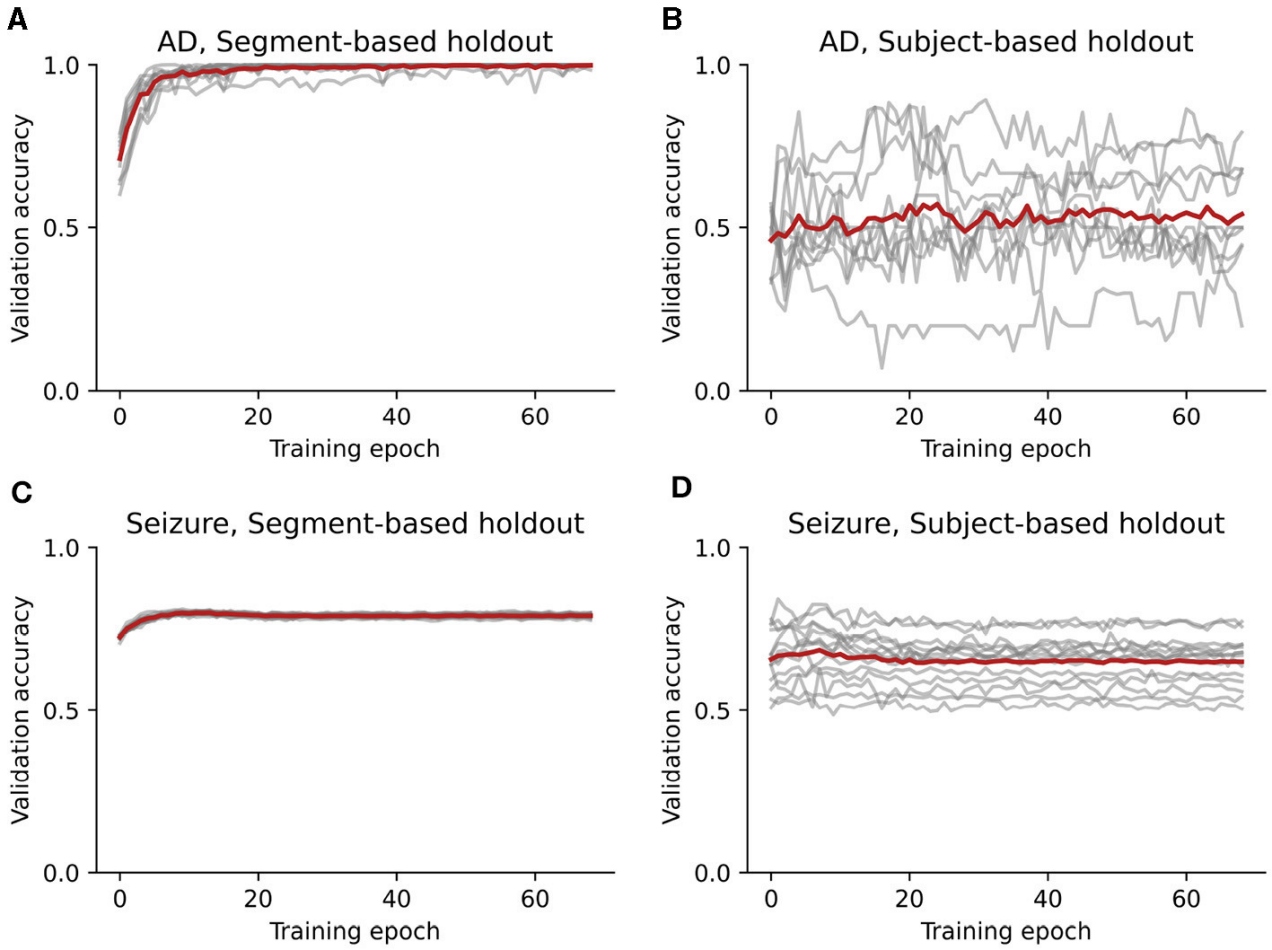
Test-set accuracy of CNN models plotted as a function of the training epoch. Gray lines show accuracy in individual cross-validation folds, and red lines show the average across folds. **(A)** Accuracy for models trained to classify Alzheimer's disease using segment-based holdout. **(B)** Accuracy for models trained to classify Alzheimer's disease using subject-based holdout. **(C)** Accuracy for models trained to identify seizures using segment-based holdout. **(D)** Accuracy for models trained to identify seizures using subject-based holdout.

To determine whether segment-based holdout leads to a biased estimate of accuracy, we first trained a CNN to diagnose Alzheimer's disease using segments of EEG. When the EEG segments were split into training and test sets without considering subject ID, the model showed nearly perfect test-set accuracy of 99.8% (99.1–100.0%) (Figure 2A). Performance quickly approached ceiling within the first 15 training epochs (Figure 3A). This high accuracy is consistent with prior studies that use segment-based holdout and report high accuracy for CNNs at identifying neurological disorders (Acharya et al., 2018b; Lee et al., 2019; Oh et al., 2020). Could this pattern of high accuracy reflect data leakage, instead of a robust and generalizable classifier?

When we used subject-based holdout, ensuring that individual subjects' data did not appear in both the training and test sets, test accuracy dropped to 53.0% (43.1–64.8%), with 95% confidence intervals that included chance performance of 50%. Performance remained low throughout the training epochs (Figure 3B). Compared with subject-based holdout, segment-based holdout significantly overestimates the model performance on previously-unseen subjects (Wilcoxon *T* = 0.0, *p* = 0.002).

#### 3.1.2 Identifying segments containing epileptic seizures

In some cases, artificial neural network models have been used to identify time-limited events within ongoing brain activity, such as epileptic seizures. Does segment-based holdout also lead to data leakage when labeling periods of time within subjects? To answer this question, we trained a CNN to classify segments of EEG data as containing an epileptic seizure or not.

When the EEG segments were split into training and test sets without considering subject ID, the model reached a high test-set accuracy of 79.1% (78.8–79.4%) (Figure 2B). Accuracy leveled out within 10 training epochs (Figure 3C). When individual subjects' data segments were restricted to appear in only the training or test set, however, accuracy fell to 65.1% (61.3–69.1%). Accuracy remained low throughout training epochs (Figure 3D). Even when the model is tasked with labeling periods of activity within subjects, segment-based holdout significantly overestimates performance on previously-unseen subjects (Wilcoxon *T* = 0.0, *p* = 0.0001).

### 3.2 Data leakage in published EEG studies

**Figure 4:**
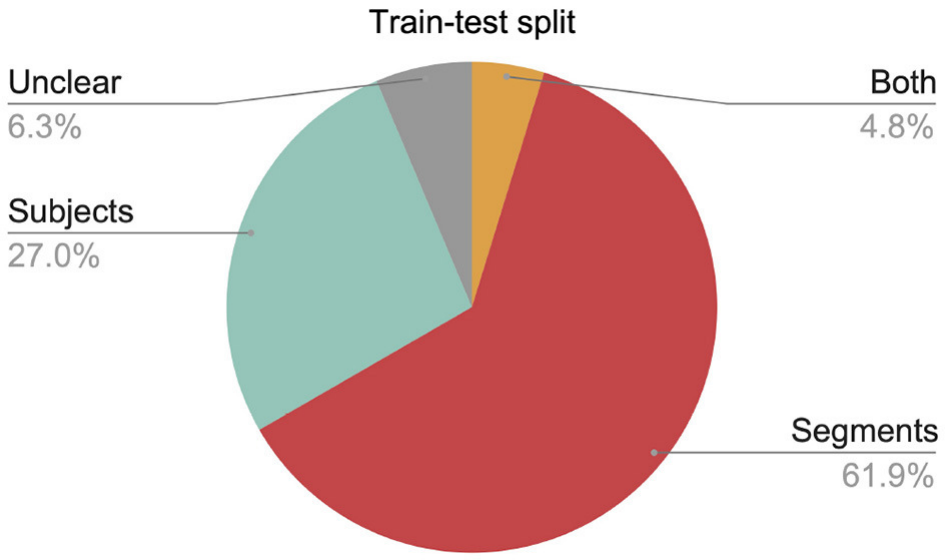
Number of studies using each type of test-split. “Segments”: Segments of EEG data were assigned to the training and test sets without regard to subject; this approach leads to data leakage. “Subjects”: Each subject's data appeared in only the training set or the test set. “Both”: Both the Subjects and Segments approaches were used in different analyses. “Unclear”: We could not determine which approach was used for train-test splits.

Do published translational EEG studies suffer from subject-specific data leakage, or do they avoid it by computing their test-set accuracy on held-out subjects? We examined the train-test split strategies in published studies that attempted to identify a clinical disorder using DNNs with EEG recordings. Out of the 63 relevant papers we found, only 17 (27.0%) unambiguously avoided this type of data leakage (Figure 4; Table 1). Leakage of subject-specific information is pervasive in the translational EEG literature.

**Table 1:** Prior translational studies using deep learning with EEG.

**Article**	**Target**	**Test split**
Ahmadi et al. (2021)	ADHD	Segments
Bakhtyari and Mirzaei (2022)	ADHD	Segments
Chang et al. (2022)	ADHD	Subjects
Chen et al. (2019a)	ADHD	Segments
Chen et al. (2019b)	ADHD	Segments
Dubreuil-Vall et al. (2020)	ADHD	Subjects
Mafi and Radfar (2022)	ADHD	Segments
Moghaddari et al. (2020)	ADHD	Segments
TaghiBeyglou et al. (2022)	ADHD	Subjects
Tosun (2021)	ADHD	Segments
Vahid et al. (2019)	ADHD	Subjects
Zhou et al. (2022)	ADHD	Unclear
Kim et al. (2018)	Alcoholism	Segments
Bi and Wang (2019)	Alzheimer's	Segments
Gkenios et al. (2022)	Alzheimer's	Both
Huggins et al. (2021)	Alzheimer's	Segments
Ieracitano et al. (2019)	Alzheimer's	Both
Kim and Kim (2018)	Alzheimer's	Subjects
Morabito et al. (2016)	Alzheimer's	Subjects
You et al. (2020)	Alzheimer's	Segments
Zhao and He (2015)	Alzheimer's	Segments
Acharya et al. (2018b)	Depression	Segments
Ay et al. (2019)	Depression	Segments
Kwon et al. (2019)	Depression	Subjects
Li et al. (2019)	Depression	Subjects
Li X. et al. (2020)	Depression	Subjects
Mumtaz and Qayyum (2019)	Depression	Segments
Uyulan et al. (2021)	Depression	Unclear
Xie et al. (2020)	Depression	Unclear
Zhang et al. (2020)	Depression	Segments
Khare et al. (2021)	Parkinson's	Segments
Lee et al. (2019)	Parkinson's	Segments
Loh et al. (2021)	Parkinson's	Segments
Oh et al. (2020)	Parkinson's	Segments
Shaban (2021)	Parkinson's	Segments
Shaban and Amara (2022)	Parkinson's	Subjects
Shi et al. (2019)	Parkinson's	Subjects
Ahmedt-Aristizabal et al. (2020)	Schizophrenia	Subjects
Chu et al. (2017)	Schizophrenia	Segments
Oh et al. (2019)	Schizophrenia	Both
Shalbaf et al. (2020)	Schizophrenia	Segments
Acharya et al. (2018a)	Seizure	Segments
Avcu et al. (2019)	Seizure	Subjects
Choi et al. (2019)	Seizure	Subjects
Daoud and Bayoumi (2019)	Seizure	Segments
Emami et al. (2019)	Seizure	Subjects
Fürbass et al. (2020)	Seizure	Subjects
Gao et al. (2020)	Seizure	Segments
Hussein et al. (2019)	Seizure	Segments
Iešmantas and Alzbutas (2020)	Seizure	Subjects
Jana et al. (2020)	Seizure	Segments
Khan et al. (2017)	Seizure	Segments
Li Y. et al. (2020)	Seizure	Segments
Liang et al. (2020)	Seizure	Segments
Raghu et al. (2020)	Seizure	Unclear
Rashed-Al-Mahfuz et al. (2021)	Seizure	Segments
Truong et al. (2018)	Seizure	Segments
Ullah et al. (2018)	Seizure	Segments
Wei et al. (2018)	Seizure	Segments
Wei et al. (2019)	Seizure	Segments
Zhao et al. (2020)	Seizure	Segments
Zhou et al. (2018)	Seizure	Segments
Bouallegue et al. (2020)	Seizure and autism	Segments

Each line in the table describes one published translational study using a DNN with EEG data. The “Target” column holds the clinical condition being classified. The “Test split” column shows the approach used to determine how the data were divided into training and test sets. “Segments”: Segments of EEG data were assigned to the training and test sets without regard to subject; this approach leads to data leakage. “Subjects”: Each subject's data appeared in only the training set or the test set. “Both”: Both the Subjects and Segments approaches were used in different analyses. “Unclear”: We could not determine which approach was used for train-test splits.

## 4 Discussion

In EEG studies using deep learning, data leakage can occur when segments of data from the same subjects are included in both the training and test sets. Here we demonstrate that leakage of subject-specific information can dramatically overestimate the real-world clinical performance of a DNN classifier. Our Alzheimer's CNN classifier appeared to have an accuracy of above 99% when using segment-based holdout, but its true performance on previously-unseen subjects was indistinguishable from chance. We found this bias in test-set performance both in a between-subjects task (identifying patients with Alzheimer's disease in Experiment 1) and in a within-subjects task (identifying segments that contain a seizure in Experiment 2). Next, we show that this type of data leakage appears in the majority of published translational DNN-EEG studies we examined. Together, these results illustrate how an improperly-designed training-test split can bias the results of DNN studies, and show that biased results are widespread in the published literature.

To be useful in a clinical setting, a diagnostic classifier must be able to identify a disease in new patients. Models trained using segment-based holdout, however, strongly overestimate their ability to perform this task. Instead, these models may learn patterns associated with individual subjects, and then associate those idiosyncratic patterns with a diagnosis. As a consequence, performance of these models drops precipitously when they are tested in new subjects, and performance is unlikely to generalize to a new dataset. When training a translational DNN classifier, the model must be tested with subjects who were not included in the training set.

Our results show that segment-based cross-validation inflates estimates of out-of-sample model performance when training on segments from resting-state EEG. However, the same principles of data leakage will apply to task-based EEG; providing a classifier with person-specific information enables it to artificially inflate performance.

Although this study focused on Alzheimer's Disease and epileptic seizures, our findings are not particular those diseases. Classification studies will overestimate model generalization whenever data from individual participants is present in both the training and test sets. Prior review articles have summarized the details and idiosyncrasies of DNN models in the context of AD (Cassani et al., 2018; Wen et al., 2020) and seizures (Rasheed et al., 2020; Shoeibi et al., 2021).

### 4.1 Data leakage in between- and within-subjects designs

We find that segment-based cross-validation overestimates performance for both between-subjects (Alzheimer's disease, Experiment 1) and within-subjects comparisons (seizures, Experiment 2). However, the magnitude of this overestimate was smaller in a within-subjects comparison (Figure 2). What leads to this difference in the size of the effect between the two tasks? In a between-subjects task, the classifier can simply associate a label with each individual participant. In a within-subjects task, however, this shortcut is not available to the model. Instead, it must learn a representation of the labels—albeit a representation that may be contaminated by multiple segments coming from the same event, or one that may be specific to a given participant.

### 4.2 Data leakage when identifying events within subjects

Instead of identifying a disease in each subject, some studies attempt to identify a diseased process in each segment of time (see Table 1). DNN models of epilepsy, for example, often aim to classify the segments of data that contain a seizure. We demonstrated in Experiment 2 that those studies are not immune to data leakage in training-test splits: the accuracy in novel subjects is strongly overestimated when the test set includes subjects who were also in the training set. This result could arise if the model uses different patterns to identify seizures in each subject.

Subject-specific studies indicate that a bespoke classifier could be trained to identify seizures in each new patient (Jana et al., 2020; Liang et al., 2020; Li Y. et al., 2020). However, this would require every patient to have a large dataset of recordings that have already been labeled, which limits the clinical utility of this approach. A more realistic approach is to train DNN models to identify events in unseen patients.

### 4.3 Data leakage in other methods

In studies which have only one observation per subject, cross-validation is trivial – single observations are simply assigned to the training or test set. However, in EEG and many other medical imagining methods, the data from each subject is routinely split into multiple segments. In this paper, we showed how data leakage can arise when a long recording is split into multiple shorter segments. However, the same principles apply to any other method that introduces statistical non-independence between the training and test sets. For example, some EEG-based DNNs treat every channel independently, and use information from each channel as a separate observation (Loh et al., 2021). Those studies are likely to suffer from substantial data leakage, since physiological sources of electrical activity appear redundantly across multiple EEG scalp electrodes (Michel and He, 2019).

These principles also apply to other medical imaging methods and classifiers. Similar patterns of “identity confounding” data leakage have been documented in studies using functional (Wen et al., 2018) and anatomical (Wen et al., 2020) MRI, optical coherence tomography (OCT) (Tampu et al., 2022), accelerometer and gyroscope recordings from smartphones (Saeb et al., 2017), audio voice recordings (Chaibub Neto et al., 2019; Tougui et al., 2021), and performance on motor tasks (Chaibub Neto et al., 2019). Furthermore, data leakage due to identity confounding is not limited to deep neural networks, and has been uncovered using random forests (Saeb et al., 2017; Chaibub Neto et al., 2019; Tougui et al., 2021) and support vector machines (Tougui et al., 2021).

### 4.4 Caveats

We find that segment-based cross-validation leads to data leakage, and this type of cross-validation is common in translational EEG studies. This conclusion mirrors results from studies examining a variety of other types of data and classifier models (Saeb et al., 2017; Wen et al., 2018, 2020; Chaibub Neto et al., 2019; Tougui et al., 2021; Tampu et al., 2022). The precise amount of data leakage and the bias that it introduces, however, are likely to differ based on the details of the experiment. For example, if a study trains a classifier to identify individual subjects with a disease, then there may be stronger bias when the study involves fewer participants (Saeb et al., 2017). The model architecture may also influence the amount of data leakage: a model that can more effectively learn subject-specific representations could show stronger bias than a model that cannot learn subject-specific patterns.

## 5 Conclusion

Data leakage occurs when EEG segments from one subject appear in the both the training and test sets. As a result, the test set accuracy dramatically overestimates the classifier's performance in new subjects. This type of data leakage is common in published studies using DNNs and translational EEG. To accurately estimate a model's performance, researchers must ensure that each subject's data is included in only the training or the test set, but not both.

## Data availability statement

Publicly available datasets were analyzed in this study. This data can be found here: EEG data for experiment 1 were provided by the Pacific Neuroscience Institute. These data are described by Ganapathi et al. (2022), and can be accessed through agreement with the authors of that study. EEG data for experiment 2 were downloaded from the publicly-available Siena Scalp EEG Database hosted on PhysioNet (https://physionet.org/content/siena-scalp-eeg/1.0.0/).

## Ethics statement

Ethical approval was not required for the study involving humans in accordance with the local legislation and institutional requirements. Written informed consent to participate in this study was not required from the participants or the participants' legal guardians/next of kin in accordance with the national legislation and the institutional requirements.

## Author contributions

GB: Conceptualization, Formal analysis, Visualization, Writing – original draft. JK: Data curation, Formal analysis, Software, Visualization, Writing – review & editing. NB: Software, Writing – original draft, Writing – review & editing. YW: Conceptualization, Software, Writing – review & editing. RG: Resources, Writing – review & editing. DM: Resources, Supervision, Writing – review & editing. SG: Funding acquisition, Supervision, Writing – review & editing. KY: Writing – review & editing. CQ: Conceptualization, Data curation, Writing – review & editing. CL: Conceptualization, Funding acquisition, Writing – review & editing.
