# Supplementary material for: Data leakage in deep learning studies of translational EEG
Source: Front Neurosci. 2024 May 3;18:1373515. doi: 10.3389/fnins.2024.1373515 (PMC11099244; doi:10.3389/fnins.2024.1373515)
Supplement: Supplementary file 1 [file Data_Sheet_1.PDF]

frontiers

Supplementary Material

1 MODEL SPECIFICATIONS

These sections provide the code necessary to produce the deep neural networks used in each experiment.

1.1 Experiment 1: AD model definition

```
AD_model = tf.keras.models.Sequential([
    tf.keras.layers.Conv1D(
        input_shape = (500, 19),
        filters = 5,
        kernel_size = (20),
        activation = 'relu',
        ),
    tf.keras.layers.MaxPool1D(pool_size = 2, strides = 2),
    tf.keras.layers.BatchNormalization(),
    tf.keras.layers.Conv1D(
        filters = 10,
        kernel_size = (10),
        activation = 'relu',
        ),
    tf.keras.layers.MaxPool1D(pool_size = 2, strides = 2),
    tf.keras.layers.BatchNormalization(),
    tf.keras.layers.Conv1D(,
        filters = 10,
        kernel_size = (10),
        activation = 'relu',
        ),
    tf.keras.layers.MaxPool1D(pool_size = 2, strides = 2),
    tf.keras.layers.BatchNormalization(),
    tf.keras.layers.Conv1D(
        filters = 15,
        kernel_size = (5),
        activation = 'relu',
        ),
    tf.keras.layers.MaxPool1D(pool_size = 2, strides = 2),
    tf.keras.layers.BatchNormalization(),
    tf.keras.layers.Flatten(),
    tf.keras.layers.Dense(20, activation = 'relu'),
    tf.keras.layers.Dropout(0.5),
    tf.keras.layers.Dense(10, activation = 'relu'),
    tf.keras.layers.Dropout(0.5),
    tf.keras.layers.Dense(2, activation = 'softmax')
])
```

1.2 Experiment 2: Seizure model definition

```
seizure_model = tf.keras.models.Sequential([
    tf.keras.layers.Conv2D(
        input_shape = (224, 224, 3),
        filters = 32,
        kernel_size = 3,
        activation = 'relu',
    ),
    tf.keras.layers.MaxPooling2D(pool_size = 2, strides = 2),
    tf.keras.layers.BatchNormalization(),
    tf.keras.layers.Conv2D(
        filters = 32,
        kernel_size = 3,
        activation = 'relu',
    ),
    tf.keras.layers.MaxPooling2D(pool_size = 2, strides = 2),
    tf.keras.layers.BatchNormalization(),
    tf.keras.layers.Conv2D(
        filters = 64,
        kernel_size = 3,
        activation = 'relu',
    ),
    tf.keras.layers.MaxPooling2D(pool_size = 2, strides = 2),
    tf.keras.layers.BatchNormalization(),
    tf.keras.layers.Conv2D(
        filters = 64,
        kernel_size = 3,
        activation = 'relu',
    ),
    tf.keras.layers.MaxPooling2D(pool_size = 2, strides = 2),
    tf.keras.layers.BatchNormalization(),
    tf.keras.layers.Flatten(),
    tf.keras.layers.Dense(256, activation = 'relu'),
    tf.keras.layers.Dense(512, activation = 'relu'),
    tf.keras.layers.Dropout(0.5),
    tf.keras.layers.Dense(2, activation = 'softmax')
])
```
